# Low spatial mobility of associated microbes along the hyphae limits organic nitrogen utilization in the arbuscular mycorrhizal hyphosphere

**DOI:** 10.3389/fpls.2025.1706684

**Published:** 2026-01-12

**Authors:** Caroline Krug Vieira, Martin Rozmoš, Michala Kotianová, Hana Hršelová, Petra Bukovská, Jan Jansa

**Affiliations:** Laboratory of Fungal Biology, Institute of Microbiology, Czech Academy of Sciences, Prague, Czechia

**Keywords:** chitin, nitrogen mineralization, microbial diversity gradient, arbuscular mycorrhizal fungal hyphae, *Rhizophagus irregularis*, microbial migration, mineral and organic nutrients, hyphosphere microbiome recruitment

## Abstract

**Bacground:**

Arbuscular mycorrhizal (AM) fungi enhance plant nutrient acquisition from soil; however, their ability to exploit organic nutrient forms in the absence of associated microbes capable of mineralization remains unclear.

**Methods:**

To test if the AM fungi carry their beneficial bacterial partners into nutrient-rich zones, we conducted three controlled experiments manipulating the microbial inputs, diversity and composition in plant–AM fungus–soil systems, ranging from open pots to semi-sterile mesocosms. We manipulated soil microbial diversity by imposing a microbial diversity gradient (complex communities fractionated by size, resulting in fractions passing through 1 µm to 1000 µm sieves) and cultivated *Andropogon gerardii* in previously sterilized substrate together with a bacterial-free *Rhizophagus irregularis*. In each experiment, ^15^N‐labeled chitin or mineral nitrogen (N) compartments were installed in the root‐free zone of each mesocosm.

**Results:**

With decreasing microbial inputs into the root-free zone, the N uptake from chitin to plants, facilitated by the AM fungal hyphae, decreased. Upon complete absence of microbes in the root-free zone, AM hyphal foraging preferences assessed by quantitative PCR indicated that exploration of the mineral N compartments was more effective than that of the chitin compartments. The AM fungal hyphae were ineffective in priming mineralization of organic N even if provided with complex soil microbiomes at a distance from the compartment.

**Conclusions:**

In summary, chitin-enriched compartments become attractive for the AM fungi only when previously mineralized by competent microbes. Such microbes, however, were not effectively transported to spatially restricted organic resources in soil via AM hyphal highways in our experiments.

## 1 Introduction

Arbuscular mycorrhizal (AM) fungi (phylum Glomeromycota) are obligate symbionts that form mutualistic associations with approximately 70% of all plant species (Brundrett and Tedersoo, 2018). These fungi rely on the provision of photosynthetically fixed carbon supplied by their plant hosts; in return, they confer a wide range of benefits (Smith and Read, 2008), including facilitating acquisition of essential nutrients from soil —such as phosphorus (P) and nitrogen (N), growth promotion, enhanced protection against pathogens, and increased tolerance to abiotic stresses such as salinity, drought, and heavy metal toxicity (Wang et al., 2017; Shi et al., 2023; Duan et al., 2024; Gatasheh et al., 2024; Boorboori and Lackóová, 2025; Zhang et al., 2025).

As the AM fungal extraradical hyphae expand, they become enveloped by a thin layer of soil, thereby creating a unique ecological niche known as the hyphosphere. This microenvironment differs markedly from both bulk soil and the rhizosphere, favoring the development of distinct bacterial communities (Gahan and Schmalenberger, 2015; Emmett et al., 2021; Yuan et al., 2021). Within this niche, the hyphae were previously hypothesized to serve as efficient conduits for bacterial dispersal, enabling colonization by such bacteria (and possibly also by other microorganisms) of larger areas than would be achievable in the absence of the fungus and overcoming the inherent limitations of individual bacterial mobility (Jiang et al., 2021; Vieira et al., 2025).

In addition to facilitating microbial dispersal, AM fungi actively modulate the composition of their hyphal-associated microbiota through continuous release of carbon-rich exudates (Luthfiana et al., 2021; Anckaert et al., 2024; Jin et al., 2024). These exudates, which contain a diverse array of signaling molecules and other metabolites, establish favorable nutritional conditions that promote proliferation and activity of various bacterial taxa (Zhou et al., 2020; He et al., 2024). Through these interactions, AM fungi are then able to acquire nutrients for their own use as well as for uptake by their host plants. The limited saprotrophic capacity of AM fungi hinders their direct access to many nutrients bound in organic compounds (Tisserant et al., 2013; Miyauchi et al., 2020; Malar et al., 2022). As a result, these fungi rely on specific bacteria or other microbes to compensate for this deficiency by mineralizing organic P and N in the soil in exchange for carbon and energy (Zhang et al., 2018; Li et al., 2023; Duan et al., 2025; Vaishnav et al., 2025).

Despite growing recognition of these interactions, important knowledge gaps persist. It is still unclear how the composition and diversity of soil microbial communities influence the mineralization of organic nitrogen, and to what extent the AM fungi — via fungal highways — facilitate the colonization of nutrient-rich microsites and the subsequent transfer of these nutrients to host plants. In this context, we conducted controlled mesocosm experiments combined with stable isotope labeling and tracing to evaluate how manipulation of microbial community composition and diversity affects nitrogen (N) mineralization from a defined organic source (chitin) and AM fungal–mediated N transfer to plants. We hypothesized that (i) the efficiency of N transfer from organic compounds depended on the presence and diversity of N mineralizing bacteria in the soil. Therefore, in mesocosms with higher microbial diversity, we expected enhanced chitin mineralization, resulting in greater plant N acquisition via hyphal networks; and (ii) because bacteria were added directly to root-free compartments in Exps. 1 and 2 but needed to migrate over longer distances in experiment 3, we hypothesized that acquisition of N from chitin would be higher in the first two experiments than in the third one unless AM fungal hyphae acted as efficient vectors, facilitating the movement of soil microbes specialized in degrading organic N. Microbial diversity was manipulated by sieving the substrate inoculum to generate communities with contrasting complexity, and the role of hyphae as potential vectors for specialized degraders was assessed by comparing microbial communities recovered from rhizosphere with those colonizing root-free compartments in Exp. 3.

## 2 Materials and methods

### 2.1 Mesocosms and microbial community inoculations

#### 2.1.1 Experiment 1

**Figure 1:**
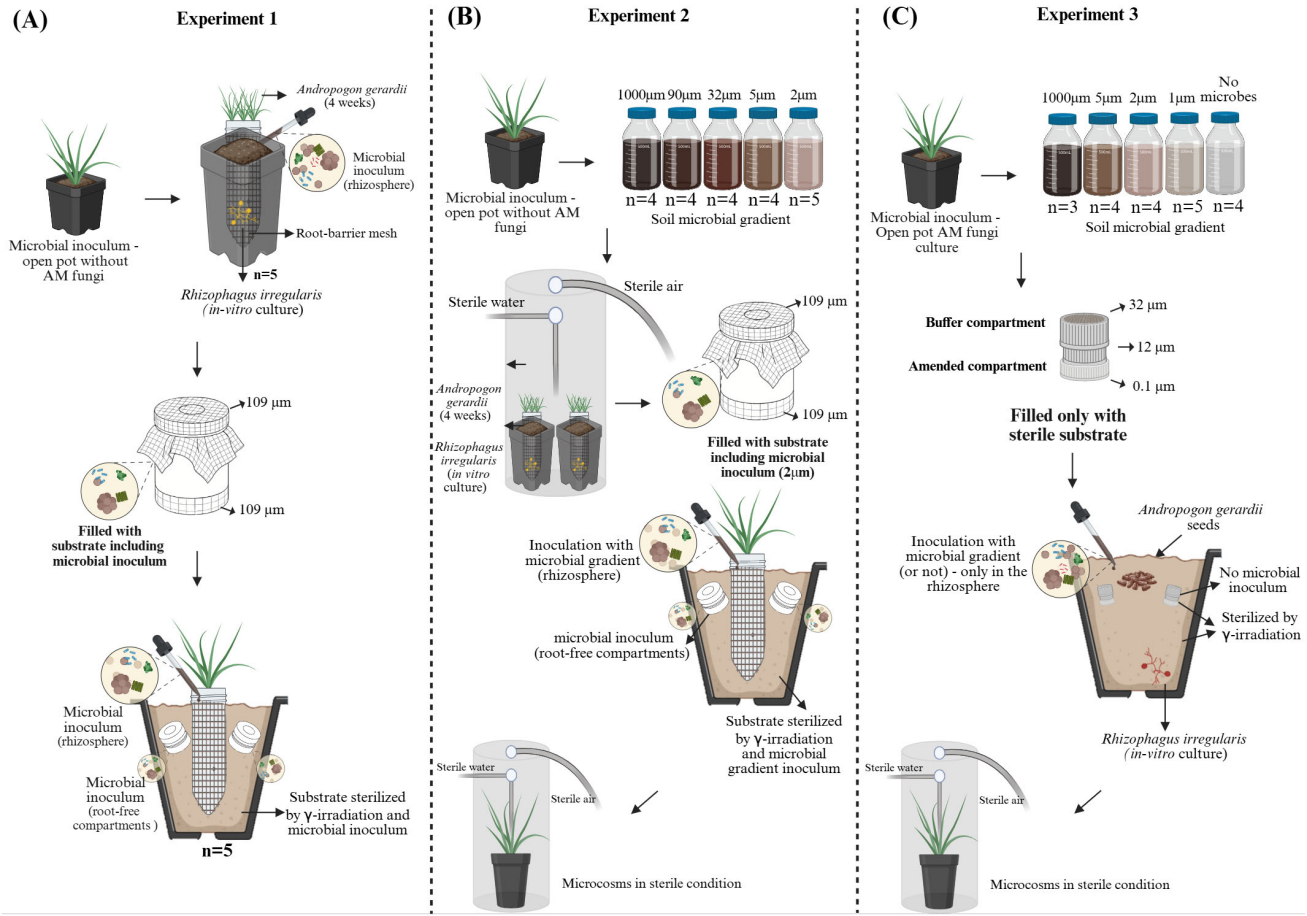
Schematic overview of the experimental designs used in the different experiments: Experiment 1 **(A)**, Experiment 2 **(B)**, and Experiment 3 **(C)**. The images were created in BioRender.com.

The experiment was conducted under non-sterile conditions. The rhizosphere and root-free compartments were inoculated with microbial communities from a previous pot culture that had hosted a non-mycorrhizal plant, thereby creating a high-diversity microbial scenario (**Figure 1**). This experimental arrangement provided a comparative baseline of complex microbial communities and served as the reference for subsequent experiments in which microbial diversity was progressively reduced to assess how community simplification affected the efficiency of ^15^N transfer to plants. Perforated plastic containers of 500 mL volume (cheese form P00718, Anelli SRL, Montanaso, Italy), lined with a 42 µm nylon mesh (Uhelon 130T, Silk and Progress, Brněnec, Czech Republic) to restrict root growth outsides of the container, were used for the pre-growth of the host plant and inoculation with AM fungus (**Table 1**, **Supplementary Figure S1A**). The containers were filled with a sterile substrate composed of 10% field soil sterilized by γ-rays (min. dose of 25 kGy), 45% autoclaved sand and 45% autoclaved granular zeolite as described previously (Bukovská et al., 2016; Gryndler et al., 2018). The substrate (further referred as “soil”) was coarsely structured, slightly alkaline (pH= 8.9 in a water slurry 1:2.5, w:v) and nutrient-poor, containing 46.5 mg kg^−1^ total P, of which 2.6 mg kg^−1^ was water-extractable (1:10 w:v, shaken for 20h, and filtered through 0.2 μm nitrocellulose mixed ester filter), as well as 0.013% and 0.22% total N and organic C, respectively (Jansa et al., 2020). The substrate was mixed (5% v:v) with previous potting soil containing rich microbial communities from nonmycorrhizal pots where leek (*Allium porrum*) was grown for more than 2 years, as specified previously (Bukovská et al., 2021). Each container was inoculated with the AM fungus *Rhizophagus irregularis* LPA9 (approximately 10000 bacteria-free spores along with associated hyphae produced in monoxenic *in vitro* systems, Řezanka et al. (2023)) and seeded with approximately 50 *Andropogon gerardii* Vitman (big bluestem) seeds, a C4 grass with a fibrous root system usually intensively colonized by AM fungi (Impastato and Carrington, 2020; Hopkins and Bever, 2024). The containers were placed into 2 L plastic pots with drainage holes, filled with the same sterile soil as above, and maintained under glasshouse conditions (Bukovská et al., 2021), with temperatures fluctuating between 17 °C and 35 °C, 14 h photoperiod combined of solar radiation and high pressure metal halide lamps, providing a minimum of 200 μmol of photosynthetically active radiation m^-2^ s^-1^. After four weeks, the plastic containers with pre-grown mycorrhizal plants were transferred to 10 L plastic pots, also with drainage holes, filled with the same sterile soil as above, and inoculated with previous potting soil containing rich microbial communities from nonmycorrhizal pots as above (5% volume by weight, see also **Supplementary Figure S1A** for a photo). At a distance of 3 cm from the plant containers, root-free compartments (polyvinylchloride (PVC) cylinders measuring 3.5 cm in diameter and 3 cm in length) were installed. The compartments were filled with 45 mL of the same soil used in the experimental pots, including the microorganisms from nonmycorrhizal inoculum, and covered at both openings with 109 µm mesh (Uhelon 63M, Silk and Progress). These compartments contained either ^15^N-chitin (>99% ^15^N atom%) prepared as described previously (Bukovská et al., 2018) or equivalent amounts of mineral N (natural abundance ^15^N, in form of sodium nitrate) and P (in form of sodium dihydrogen phosphate). This is because our chitin purified from zygomycetous cell walls always contains significant amount of P (possibly in form of inorganic polyphosphates). Each compartment thus received nutrient amendments containing 0.78 mmol N and 96.3 µmol P. Each pot included one compartment containing ^15^N-chitin and one mineral nutrient (NP) control compartment. A completely randomized experimental design was used, and the open pots were maintained in the glasshouse for an additional eight weeks. Some of the results of this experiment were previously published by Bukovská et al. (2021, experiment 1). We only make use of the five pots from this experiment here, namely those inoculated with the AM fungus, amended with ^15^N-chitin, and provided with normal (not elevated) N nutrition.

**Table 1:** Comparison of the parameters and methodological differences among three mesocosm experiments included in this research.

Experiment identity	Experiment 1 (Bukovská et al., 2021)	Experiment 2	Experiment 3
Environment	Open pots, glasshouse	Semi-sterile mesocosms, glasshouse	Semi-sterile mesocosms, glasshouse
Pot size	10L	10L	7.5L
Potting soil	Standardized soil:zeolite:sand 1:4.5:4.5	Standardized soil:zeolite:sand 1:4.5:4.5	Standardized soil:zeolite:sand 1:4.5:4.5
Soil processing	Mixing individual sterile components and adding mock microbial communities in the lab	Mixing individual sterile components and adding mock microbial communities in the lab	Pre-assembly of pots and gamma-irradiation of whole pots, then only adding microbes into the middle of the pots, under the seeds
Plant pre-growth (2L pots with root compartment)	Yes (4 weeks)	Yes (4 weeks)	No (direct planting into pre-assembled and irradiated pots)
Duration of the experiment with root-free compartments included	8 weeks	4 weeks	9 weeks
AM fungi inoculum	*in vitro* produced (LPA9)	*in vitro* produced (LPA9)	*in vitro* produced (LPA9)
Microbial inputs	Mock inoculum (potting soil from a previous culture from glasshouse without AM fungi), added everywhere, including the root-free compartments	Soil filtrate (2 µm) added to the root-free compartments. Microbes filtered through different sieves/filters added to the remaining soil (rhizosphere and root-free compartments)	Microbes filtered through different sieves/filters added to the rhizosphere only, the rest completely sterile at the beginning
Size of the opening in the meshes between roots and compartments	42 µm (rhizosphere delimitation) + 109 µm (root-free compartment delimitation)	42 µm (rhizosphere delimitation) + 109 µm (compartment delimitation)	32 µm (rhizosphere delimitation) + 12 µm (compartment delimitation)
Volume of labeled compartment	45 mL	45 mL	10.6 mL
Amount of ^15^N chitin added per labeled compartment	195 mg	202 mg	40 mg
Nitrogen form added to mineral nutrient (NP) compartment	Nitrate (NO_3_^-^)	Nitrate (NO_3_^-^)	Ammonium (NH_4_^+^)
Pot compartments analyzed	Rhizosphere, chitin (^15^N-labeled) and mineral nutrient (NP) compartments	Rhizosphere, chitin (^15^N-labeled) and mineral nutrient (NP) compartments	Rhizosphere, chitin (^15^N-labeled) and mineral nutrient (NP) compartments

#### 2.1.2 Experiment 2

Mesocosms with distinct microbial communities were established under semi-sterile conditions to assess how the simplification of soil microbial communities, including in root-free compartments, affected the efficiency of ^15^N transfer to plants (**Figure 1**). To generate different diversity levels, 250 g of soil collected from a non-mycorrhizal inoculum pot planted in leeks and maintained under glasshouse conditions was suspended in 1 L of water, manually agitated, and passed sequentially through sieves and membrane filters of decreasing mesh size — 1000 µm (n = 4), 90 µm (n = 4), 32 µm (n = 4), 5 µm (n = 4), and 2 µm (n = 5) — producing approximately 150 mL of filtrate per treatment and resulting in a total of 21 mesocosms (**Table 1**; **Supplementary Figure S1B**). This method reduced the abundance of different soil microbial groups while simultaneously modifying community composition (Bradford et al., 2002; Wagg et al., 2014).

The previously described 2 L plastic pots were filled with sterile soil. Inside each pot, a 500 mL perforated container (cheese mold lined with a 42 µm nylon mesh) was filled with sterile soil combined with the respective microbial filtrate (25 mL per container) and *R. irregularis* inoculum (10000 spores + hyphae, mixed throughout the container). Approximately fifty A*. gerardii* seeds were added 5–10 mm below the surface of each container. After setup, the pots were enclosed within Poly (methyl 2-methylpropenoate, PMMA) cylinders (30 × 66 cm), which were tightly sealed to their bases with screws to ensure sterile airflow and water entry exclusively from the top (see **Supplementary Figure S1** for photos). Aeration (150 mL min^-1^) was provided through Whatman filters (No. 10463607), and watering was performed with deionized and autoclaved water. An aeration system, connected immediately after setup, used a bubbler flask with water to humidify the internal air. Prior to use, all containers and equipment — including hoses, cylinders, and pots — were disinfected with a 1:200 sodium hypochlorite solution, thoroughly rinsed with running water, and completely dried.

After four weeks, the pots were removed from the cylinders, and the plastic (central) containers and the remaining pots were separated. New mesocosms were established in larger 10 L pots using 1.5 kg of recycled substrate from the previous cultures and approximately 7 kg of additional sterile substrate prepared with the same microbial filtrate (50 mL filtrate per liter substrate) as inserted previously to each microbial treatment. The cylinders containing the pre-cultivated plants were inserted into the middle of the large pots (see **Supplementary Figure S1C** for a photo). In these pots, root-free compartments were installed, consisting of PVC cylinders as in Exp. 1 (3.5 cm in diameter × 3 cm in length) filled with 45 g of sterile substrate inoculated with 2 µm microbial filtrate. These compartments were covered with a 109 µm mesh and received 202 mg of chitin labeled with ^15^N (>99% atom%) or corresponding amount of mineral N (0.85 mmol) and P (58.6 μmol) per compartment in nitrate and orthophosphate forms, respectively. Each pot included one compartment containing chitin and one with mineral N and P. The pots were relocated back to the PMMA cylinders to maintain semi-sterile conditions. A completely randomized design was used and kept in the glasshouse for an additional 28 days (from February to March 2020) under supplemental lighting with a 14 h photoperiod and controlled temperatures (averaging 22.2 ± 3.2 °C), while temperatures inside the PMMA cylinders ranged from 17.7 to 38.9 °C (monitored on two separate units).

#### 2.1.3 Experiment 3

In this study, we also investigated how simplification of the microbial community affected ^15^N transfer efficiency to plants. Notably, in contrast to Experiment 2, no microbial filtrate was introduced into the root-free compartments (**Figure 1**). This approach allowed us to directly assess whether microorganisms residing in the rhizosphere could access these compartments via AM fungal hyphae and mineralize the provided nutrients. Mesocosms were established directly in 7.5 L plastic pots with drainage holes (**Table 1**; **Supplementary Figure S1E**). These pots were filled with the same sterile soil as above and the root-free compartments were included right from the beginning. These compartments consisted of polyethylene terephthalate (PET) cylinders (4 cm in diameter × 6 cm in length) with two chambers (see **Supplementary Figures S1G–H** for a photo). The amendment (or labeling) chamber of the root-free compartment was filled with 10 g of soil and supplemented either with 40 mg of ^15^N-labeled chitin (> 99% atom% ^15^N) containing 0.15 mmol N and 11.7 μmol P or with the same amounts of N (natural ^15^N abundance) and P in forms of ammonium chloride and sodium dihydrogen phosphate, respectively. After assembling the mesocosms, they were individually packed in plastic bags and sterilized by γ-rays providing a minimum dose of 25 kGy. This also was the reason to change the material for manufacturing the root-free compartments – the PVC would not withstand such a high irradiation dose without structural damage.

The manipulation of microbial diversity and composition was achieved by fractionating soil communities based on size, using a sequence of sieves and progressively smaller mesh filters, similarly to Experiment 2 above. Microbial filtrates were obtained from 400 g of previous potting soil collected from a mycorrhizal inoculum pot (*R. irregularis* LPA9) planted in leeks and maintained in a glasshouse for 2 years. The substrate was suspended in 2 L of water, manually agitated with a spatula, and sequentially filtered through sieves, followed by processing with filter membranes of 5 µm, 2 µm, and 1 µm pore sizes. Treatments consisted of five microbial inoculations based on the filter size, 0 µm (no microbial inputs, n=4), 1 µm (n=5), 2 µm (n=4), 5 µm (n=4) and 1000 µm (n=3), totaling 20 mesocosms established. Mesocosms containing microorganisms received inoculation with 100 mL of the corresponding microbial filtrate to the center of the pots, just beneath the seeds, whereas those with no intended microbial inputs received 100 mL sterile water. All mesocosms were inoculated with *in vitro* produced cultures of *R. irregularis* (10000 spores and hyphae per pot) and sown with surface-sterilized seeds of *A. gerardii*, and then placed inside disinfected PMMA cylinders. These cylinders were sealed and secured to bases with screws to ensure the exclusive entry of sterile air and water, as described in Experiment 2 (see **Supplementary Figure S1F** for a photo). The experiment was conducted in a glasshouse from January to March 2023, lasting a total of 9 weeks, under a completely randomized design. During the experimental duration, the mesocosms were provided with supplemental lighting (14 h photoperiod) and a controlled ambient temperature of 19.4 ± 1.8 °C. The temperature within the PMMA cylinders ranged from 16.9 to 41.7 °C (monitored on two separate units).

### 2.2 Arbuscular mycorrhizal fungal inoculum

The AM fungus *Rhizophagus irregularis* LPA9 (=BEG236) was cultivated *in vitro* over six months in compartmented bioreactors associated with *Cichorium intybus* Ri-T-DNA transformed roots. The bioreactors were filled with MSR liquid medium (Cranenbrouck et al., 2005; Rozmoš et al., 2022) with four-fold elevated P concentration (Řezanka et al., 2022, Řezanka et al., 2023). The roots were floating above the liquid medium on a 42 μm nylon mesh, which allowed fungal hyphae to penetrate into the MSR medium while preventing root contact. Each pot was inoculated with fresh hyphal biomass and spores (none of the experiments described above included a nonmycorrhizal treatment).

### 2.3 Sample collection and processing

Upon harvest, shoot and root biomass was harvested from each pot. The biomass samples were then dried at 65 °C for 3 days to determine dry weights. Representative samples of the potting soil were collected from the root compartment (rhizosphere), and the root-free compartments (both ^15^N-chitin enriched and mineral N and P supplemented) from all pots. All samples were dried at 65 °C, pulverized using a ball mill MM200 (Retsch, Haan, Germany) at 25 Hz for 2 min and further processed for isotopic and molecular analyses.

### 2.4 Isotopic analyses

The total N concentrations, and the stable isotopic composition of N were measured using a Flash 2000 CN analyzer equipped with ZeroBlank autosampler and coupled with a Delta V Advantage isotope ratio mass spectrometer via ConFlo IV interface (ThermoFisher Scientific, Waltham MA, USA). For biomass samples, aliquots of approximately 2 mg were processed, and for soil samples, aliquots of approximately 20 mg were used, packed in pure tin capsules.

### 2.5 Molecular quantification of prokaryotes and AM fungus

DNA was extracted from soil samples using DNeasy PowerSoil kit (Qiagen, Venlo, the Netherlands), following the manufacturer’s recommendation, upon spiking internal DNA standard (ISC, 20 billion copies) into each sample prior to extraction as detailed previously (Thonar et al., 2012).

Abundance of *R. irregularis* and prokaryotes in the different samples were measured by quantitative real-time PCR (qPCR; **Supplementary Table S1**) using *intra* primers and TaqMan probe described by Thonar et al. (2012) and Eub primers described by Lane (1991) and listed by Dudáš et al. (2022) and **Supplementary Table S1**. Results of the different qPCR assays were corrected for the DNA losses upon extraction by using the ISC recovery measured for each individual sample as described by Thonar et al. (2012). Each qPCR assay was first calibrated with the product of endpoint PCR performed with the corresponding primers on the DNA extracted from five different soil samples. DNA concentration in the amplicon samples was measured using the Quant-iT PicoGreen double-stranded-DNA assay (Thermo Fisher Scientific, Waltham MA, USA) on a plate reader (Infinite 200 Pro; Tecan, Männedorf, Switzerland). The qPCR quantification was conducted in 96-well plates, with a final reaction volume of 20 μL. Depending on whether the primer sets were designed in conjunction with TaqMan (hydrolysis) probes (i.e., the *intra* and ISC markers), which would be double labeled with fluorescein as a fluorophore and BHQ1 as a black hole quencher, or not (the latter was the case of Eub primers), reaction mixtures were prepared using two master mixes. Specifically, we utilized the Luna universal probe qPCR master mix (M3004) for assays involving a probe and the Luna universal qPCR master mix (M3003) for those without a probe, both purchased from New England Biolabs (Ipswich MA, USA). Fluorescence data were recorded in the SYBR green/fluorescein color channel. All analyses were carried out using the LightCycler 480 II instrument (Roche, Rotkreuz, Switzerland).

### 2.6 Analyses of prokaryotic communities

Prokaryotic community profiles were generated from the rhizosphere and from the root-free compartments (i.e., ^15^N chitin and mineral NP - added) samples. The amplicons generated by the primers 515-IL/806-IL for 16S rRNA V4 region of prokaryotes were produced in technical triplicates using DNA-free PCR polymerase (TopBio, Vestec, Republic), double indexed using Nextera XT indexes coupled to Illumina sequencing adapters, and sequenced using the Illumina 2×300 platform at the Joint Microbiome Facility (Vienna, Austria) as described previously (Caporaso et al., 2011; Dudáš et al., 2022; **Supplementary Table S1**). Raw sequences from all three experiments were processed together. First, they were demultiplexed and sequencing adapter-trimmed, potential chimeras removed, primers removed, quality filtered and clustered at 97% similarity levels in the Seed 2 software (Větrovský et al., 2018) as described previously (Dudáš et al., 2022). Taxonomic assignment of sequences was then based on the SILVA database. Non-target sequences identified as chloroplasts, mitochondria, or eukaryotes were filtered out. Samples were then rarefied to equal sequencing depth (25000 reads per sample), potential chimeras removed, and clustered at 97% similarity level to yield operational taxonomic units (OTUs) and the most abundant sequences per OTU were re-identified again. Relative abundances of the different microbial taxa (clumped at genus or higher, up to the phylum, levels instead of 97% similarity levels) per sample were then used for subsequent statistical analyses. Raw data were deposited in NCBI Sequence Read Archive (SRA) under BioProject PRJNA1289392.

### 2.7 Statistical analyses

All data analyses were performed using R version 4.4.2 (R Core Team, 2021) unless specified otherwise, using “vegan” and “emmeans” packages and statistical significance threshold set at *p*-value < 0.05. Normality of distribution and homogeneity of variance were assessed using Shapiro–Wilk and Levene’s tests, respectively. ANOVA test was used and first fit linear regression model and then Tukey *post-hoc* multiple pairwise comparisons between groups were performed using the estimated marginal means. One-way ANOVA was used to determine the effects of inoculation with different microbial filtrates and nutrient enrichment with ^15^N-labeled chitin or mineral NP on plant parameters, qPCR data and microbial diversity across the different experiments. The effects of treatments and compartments on prokaryotes relative abundance for experiments 1, 2 and 3 were tested by two-way ANOVA. Beta diversity analysis assessed dissimilarity among compartments and microbial input treatments using Permutational multivariate analysis of variance (ADONIS) with 10000 permutations and non-metric multidimensional scaling (NMDS). Prokaryotic community analyses were carried out in Canoco 5.15 software (Ter Braak and Šmilauer, 2018). A principal component analysis (PCA) was first applied to explore the natural variation in community composition across samples without imposing explanatory variables. Subsequently, redundancy analysis (RDA) was performed to assess the influence of specific factors, including: 1. the influence of placing of the microbial inputs into the different mesocosms, 2. filter size for obtaining microbial inoculum (with unfractionated soil inoculum equated to 1000 µm sieve for the purpose of this analysis), and 3. the effect of root-free compartment quality (organic vs. mineral NP) on the community composition. Only taxa (either prokaryotic genera – 270 taxa, or phyla – 24 taxa) with at least five occurrences in the dataset (138 samples) and with relative abundance exceeding 0.25% in at least one sample were considered for the above analyses.

## 3 Results

### 3.1 Plant parameters

**Figure 2:**
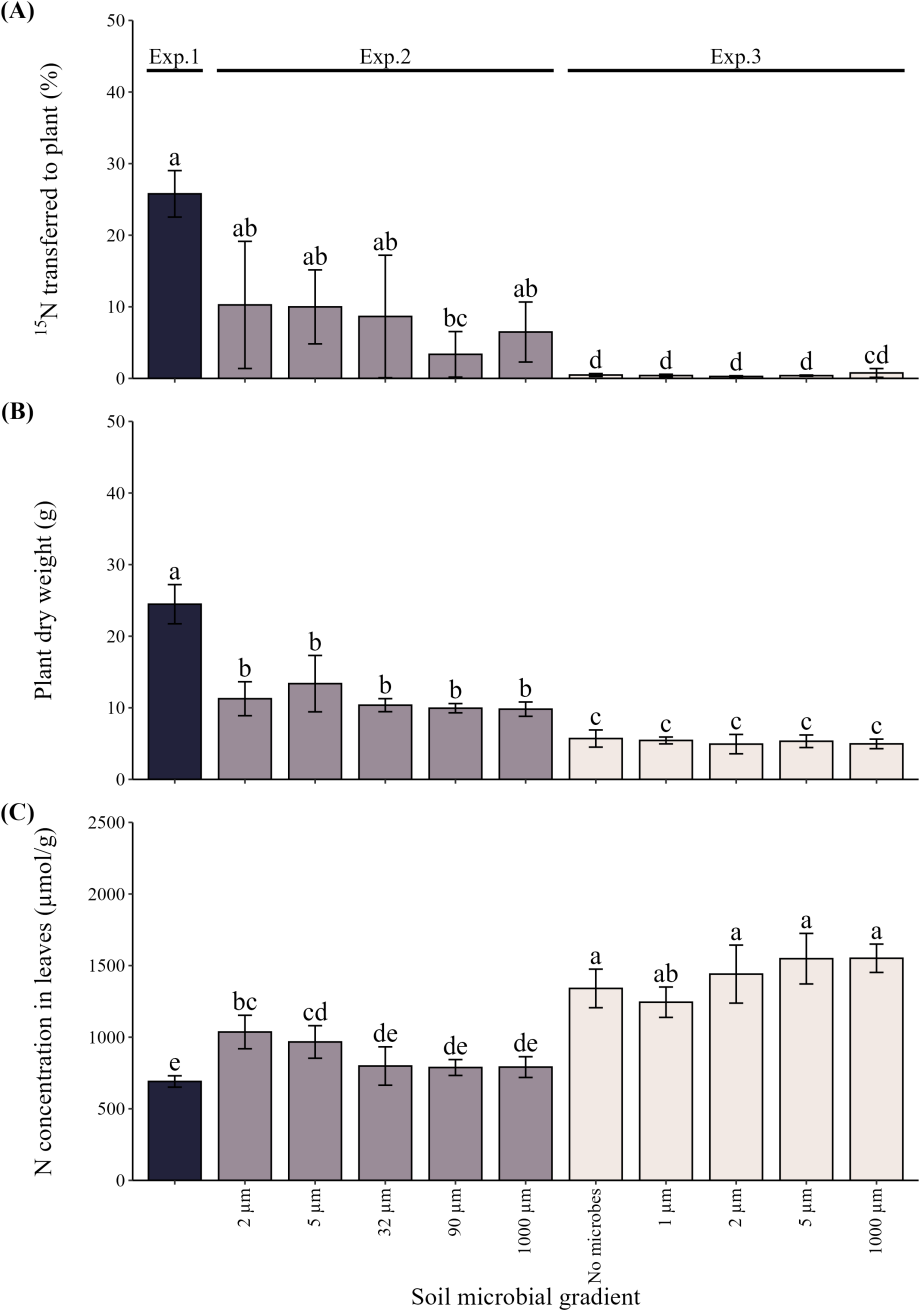
The acquisition of ^15^N from labeled chitin administered in the root-free compartment **(A)**, plant biomass **(B)** and nitrogen (N) concentrations in the plant leaves **(C)** in the different experiments and microbial input treatments (mesh sizes are indicated). Different letters indicate statistically significant differences between experiments and treatment means, determined by one-way ANOVA (*p* < 0.05) followed by Tukey’s *post hoc* test. Means ± standard errors are shown.

The fraction of ^15^N inputs transferred to plants from isotopically labeled chitin ranged from 3.4% (90 µm) to 10.3% (2 µm) in Exp. 2 (**Figure 2A**; **Supplementary Table S2**). Compared to Exp. 1, only the microbial community obtained by filtration through 90 µm sieve exhibited a significantly lower value of ^15^N transfer to plants (**Figure 2A**). In Exp. 3, the fraction of ^15^N transferred to plants remained low for all microbial input treatments, ranging from 0.26% (2 µm) to 0.76% (1000 µm), with all the treatment mean values being significantly lower than those encountered in Exps. 1 and 2 except the treatment receiving microbes passed through 1 mm sieve (**Figure 2A**). This means there were nearly two orders of magnitude differences between the Exps. 1 and 3 with respect to efficiency of plant ^15^N uptake from isotopically labeled chitin.

Plant biomass production differed between the different experiments by a factor of four, with the Exps. 2 and 3 carried under semi-sterile conditions showing generally less biomass production than plants in the Exp. 1 carried out in open pots (*p* < 0.001, **Figure 2B**; **Supplementary Table S2**). Contrary to the trends observed for other plant parameters, leaf N concentrations showed the opposite trend to plant biomass and ^15^N transfer from chitin to the plants, with the lowest values found in Exps. 1 and 2 as compared to Exp. 3 (**Figure 2C**; **Supplementary Table S2**). The total amount of N in the leaves of *Andropogon* plants was highest in Exp. 1 (8.9 mmol), followed by Exp. 3, where the treatment means ranged from 4.9 mmol N (2 µm) through 6.1 (5 µm) but did not significantly differ from each other within the Exp. 3. Treatment means in Exp. 2 ranged from 3.5 mmol N (90 µm) through 4.9 mmol N (2 µm), and only the means from the 90 µm and 1000 µm treatments were lower than any of the treatments in Exp. 3 (data not shown).

### 3.2 Development of the AM fungus in soil

**Figure 3:**
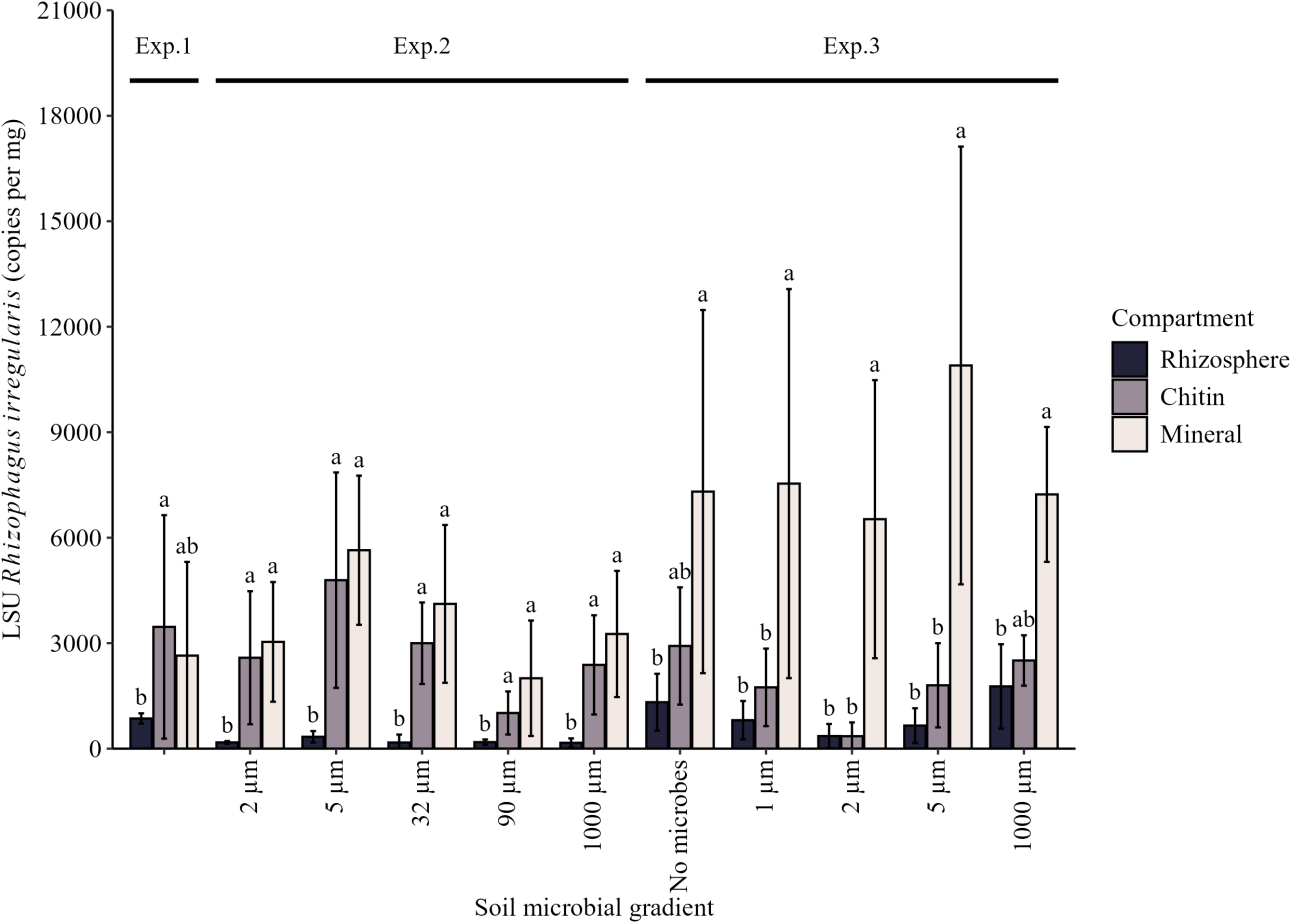
Quantification of the development of *R. irregularis* in the soils collected from rhizosphere, ^15^N chitin- and mineral NP-amended root-free compartments, as assessed by quantitative real-time PCR using specific marker (primers with a TaqMan probe) targeting the large nuclear ribosomal subunit (LSU) gene of the ribosomal operon. Lowercase letters compare values between rhizosphere and the two root-free compartments within each triplet (a microbial manipulation treatment within each single experiment) separately. Different letters indicate statistically significant differences, determined by one-way ANOVA (*p* < 0.05) followed by Tukey’s *post-hoc* test. Means ± standard errors are shown.

The qPCR revealed significant differences in *R. irregularis* development depending on the microbial and nutrient inputs in the different mesocosm compartments (**Figure 3**; **Supplementary Table S3**). In Exp. 1, the chitin-containing compartment significantly stimulated AM fungal development compared to the rhizosphere. In Exp. 2, the abundance of *R. irregularis* was higher in both of the root-free compartments than in the rhizosphere, rather a similar pattern as observed in Exp. 1 (**Figure 3**). In Exp. 3, however, only the compartments enriched with mineral NP significantly promoted AM fungal development, whereas the AM fungal abundance in both the rhizosphere and chitin compartments remained lower than that across all microbial manipulation treatments, although the absolutes numbers were at least as high as in the Exps. 1 and 2 (**Figure 3**).

### 3.3 Quantification and diversity of prokaryotic communities

**Figure 4:**
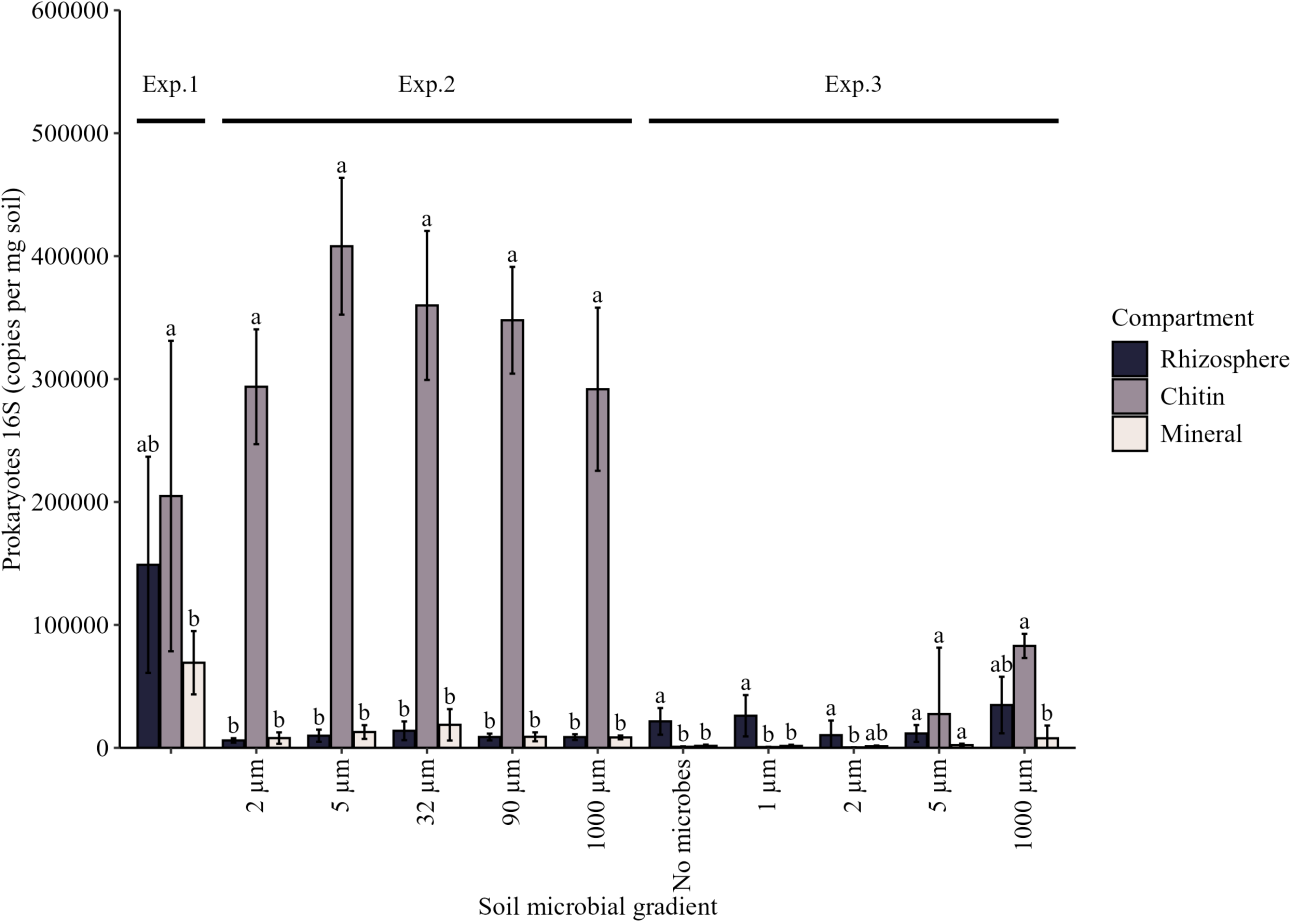
Quantification of prokaryotic abundance determined by quantitative real-time PCR targeting 16S rRNA gene in the different mesocosm compartments in the three experiments. Lowercase letters compare values between the rhizosphere, ^15^N labeled chitin- and mineral NP-amended compartments within each triplet (a microbial manipulation treatment within each single experiment) separately. Different letters indicate statistically significant differences, determined by one-way ANOVA (*p* < 0.05) followed by Tukey’s *post-hoc* test. Means ± standard errors are shown.

Prokaryotic abundance across different microbial manipulation treatments in the different experiments was generally higher in Exp. 1 than in the other two experiments. Namely, the rhizosphere and the NP compartments in Exp. 1 were both settled with more abundant bacterial communities than the respective compartments in the other two experiments (**Figure 4**; **Supplementary Table S3**). Prokaryotic abundance in the chitin-amended compartment was high in Exps. 1 and 2 and generally limited in Exp. 3 (**Figure 4**). Across all the microbial manipulation treatments in Exp. 2, prokaryotic abundance was significantly higher in compartments enriched with chitin compared to those supplemented with mineral NP and to the rhizosphere (**Figure 4**). In Exp. 3, the results were more stratified, in treatments with no added microbes and with those passing through 1 µm filter, the highest prokaryotic abundance was recorded in the rhizosphere as compared to any other compartment. In the treatment with microbes passing through a 2 µm membrane, fewer prokaryotes were detected in the chitin compartment than in the rhizosphere. In contrast, when microbes were filtered through a 5 µm membrane, prokaryotic abundance did not differ among compartments. And finally, more microbes were detected in the chitin compartment than the mineral NP compartment in the treatment added with microbes passing through 1 mm sieve (**Figure 4**).

**Figure 5:**
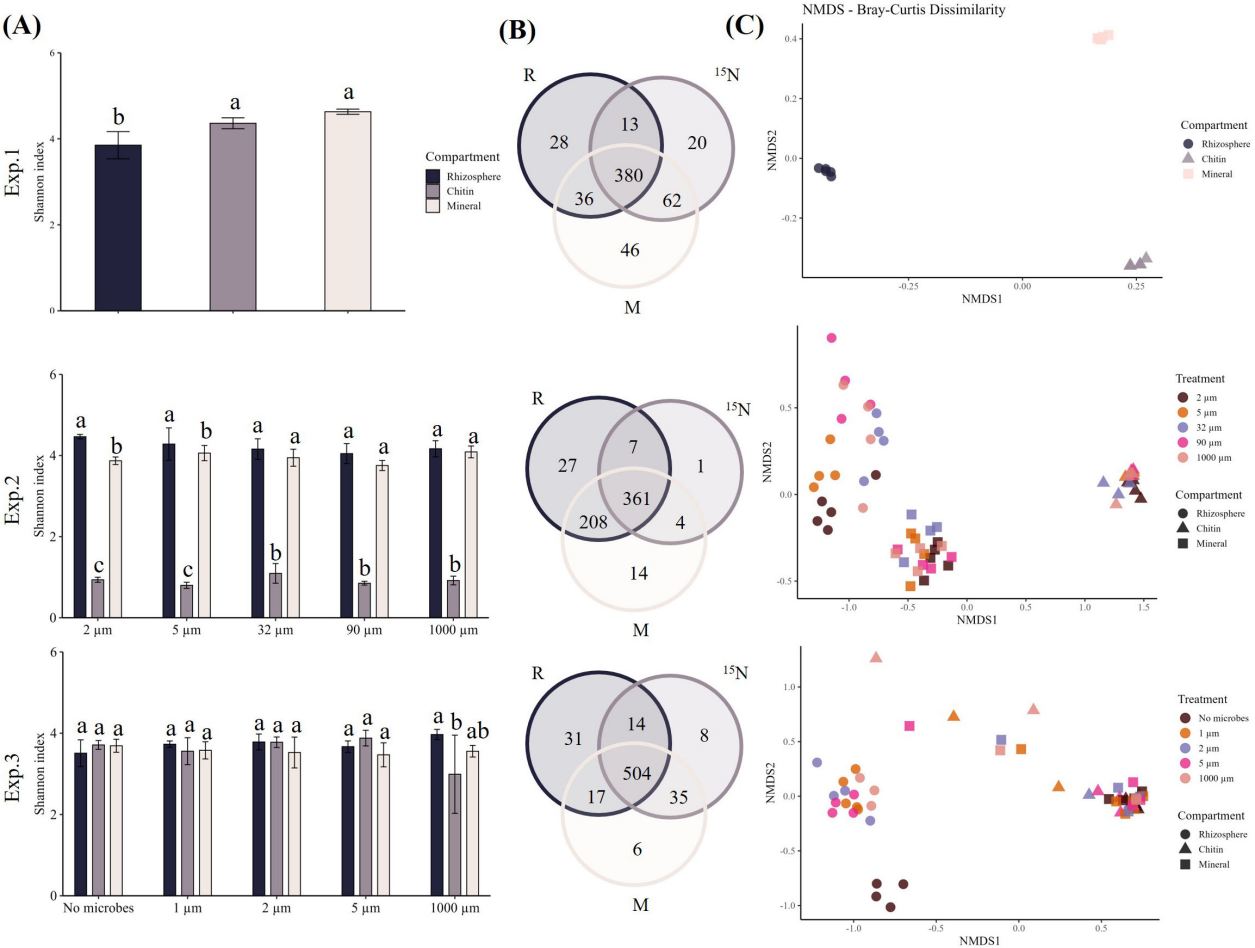
Analysis of prokaryotic diversity and composition based on 16S rRNA gene amplicon sequencing and assignment to genera in Experiment 1 (n=5), Experiment 2: 2 µm (n=5), 5 µm (n=4), 32 µm (n=4), 90 µm (n=4), and 1000 µm (n=4), and Experiment 3: No microbes (n=4), 1 µm (n=5), 2 µm (n=4), 5 µm (n=4), 1000 µm (n=3). **(A)** Alpha diversity is represented by Shannon indices, comparing diversity between compartments in each treatment separately. Statistical significance was assessed using one-way ANOVA (*p* < 0.05, followed by the Tukey's *post hoc* test); **(B)** Venn diagrams of unique and shared prokaryotic genera in rhizosphere (R), ^15^N labeled chitin (^15^N) and mineral NP root-free compartments (M) within each of the experiments; **(C)** Analysis of beta diversity of the microbial communities among microbial manipulation treatments and mesocosm compartments within each of the experiments using multidimensional scaling.

With respect to prokaryotic community diversity, substrate amendment either with chitin or with mineral N and P significantly increased microbial community diversity compared to the rhizosphere in Exp. 1 (**Figure 5A**; **Supplementary Table S4**). Yet, no significant difference was observed between the two root-free compartments in the same experiment. In Exp. 2, the compartment with ^15^N labeled chitin showed markedly lower microbial diversity than the other two compartments across all microbial manipulation treatments (**Figure 5A**). Additionally, compartments receiving the 2 and 5 µm microbial filtrates also showed lower microbial diversity in the mineral NP compartment compared to the rhizosphere (*p* < 0.001, **Figure 5A**). On the other hand, except for the treatment inoculated with the 1000 µm microbial filtrate in Exp. 3, where the ^15^N labeled chitin compartment showed reduced diversity compared to the rhizosphere (*p* = 0.03, **Figure 5A**), no significant differences in prokaryotic diversity were observed in the Exp. 3. A substantial number of prokaryotic genera was shared between all the three compartments in all three experiments (**Figure 5B**).

**Figure 6:**
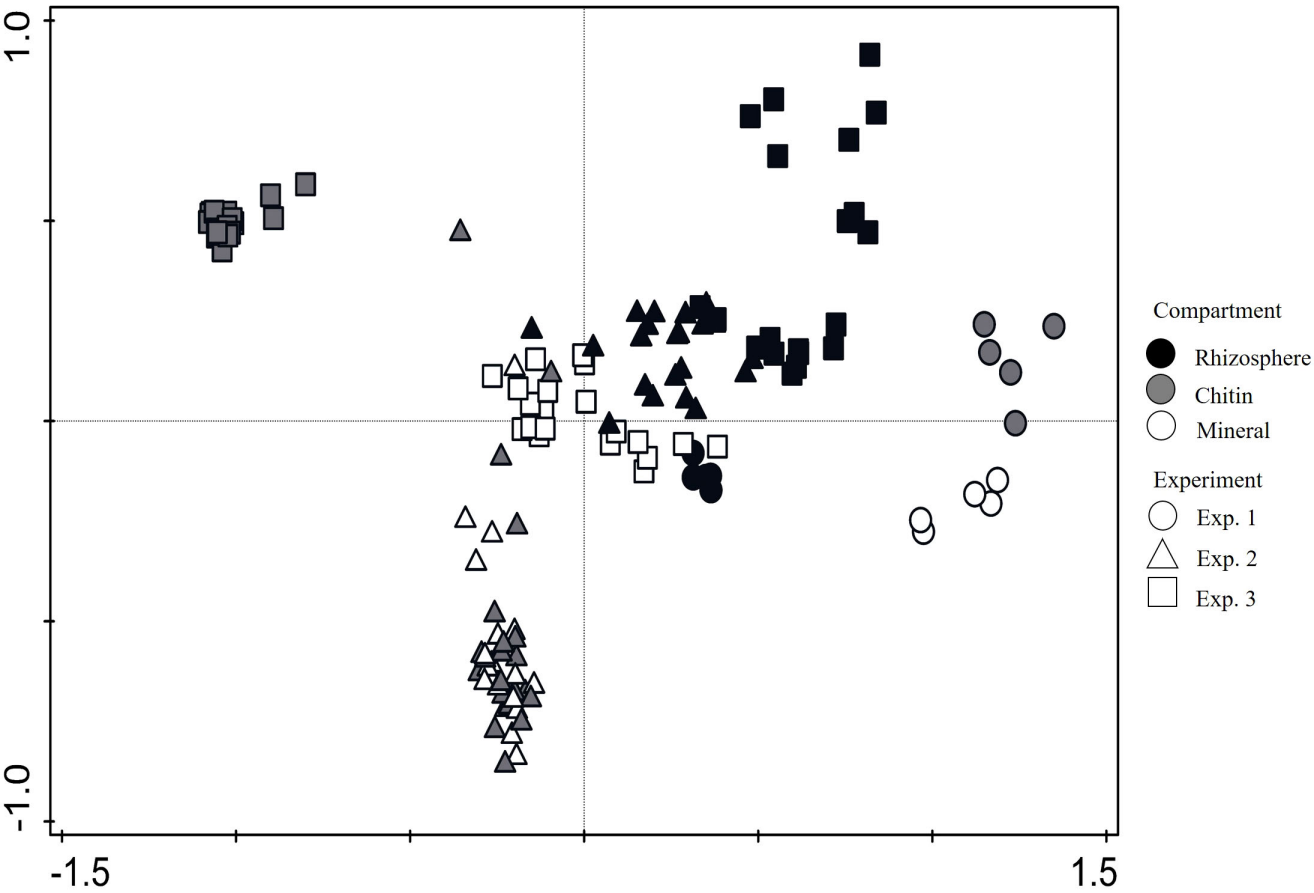
Principal component analysis of bacterial phylum-level community composition across mesocosm compartments in Experiments 1, 2, and 3, based on 16S rRNA gene amplicon sequencing. Each point represents an individual sample, and the different shades of grey stand for the different compartments: rhizosphere (black), ¹^5^N−labeled chitin (gray), and mineral NP (white). Different shapes denote experiments: circles for Experiment 1, squares for Experiment 2, and triangles for Experiment 3.

Bacterial beta diversity, assessed by PERMANOVA using Bray–Curtis dissimilarity and represented by NMDS, was significantly affected by microbial manipulation in Exps. 2 and 3, and by the specific form of N amendment in the root-free compartments in all three experiments (Exp. 1, *p* < 0.001, stress value: < 0.05; Exp. 2, *p* < 0.001, stress value: 0.05; Exp. 3, *p* < 0.001, stress value: 0.07) (**Figure 5C**). The PCA, integrating data from all three experiments, revealed distinct clustering of bacterial communities across the different compartments (**Figure 6**). Rhizosphere samples consistently clustered close to each other, regardless of the experiment, indicating a stable microbial community structure associated with this compartment. In contrast, the mesocosm compartments enriched with either ^15^N labeled chitin or mineral NP in Exp. 1 clustered apart from each other, whereas those from Exp. 2 did overlap to a great extent (**Figure 6**). In Exp. 3, the differently amended root-free compartments showed a very clear distinction, clustering far apart from each other (**Figure 6**).

The RDA revealed significant differences in the bacterial community composition both at the phylum and at the genus levels across compartments and experiments. Each experiment exhibited different dominant phyla, indicating distinct response patterns to the specific experimental conditions (**Supplementary Table S5**). Notably, Dependentiae, one of the most abundant phyla in Exp. 1, was also highly represented in the ^15^N labeled chitin compartment across the different experiments (*p* < 0.001). Similarly, Actinobacteriota, predominant in Exp. 2, was highly abundant in the chitin-amended compartments across the experiments (*p* < 0.001). Conversely, the most abundant phyla in Exp. 3, which included Acidobacteriota, Crenarchaeota, Firmicutes and Methylomirabilota, were showing higher abundance in the mineral NP compartment across the different experiments (*p* < 0.001). Although each experiment and compartment harbored abundant and often distinct microorganisms, a subset of taxa was shared between the mineral NP compartment and the rhizosphere (**Supplementary Table S4**). Focusing on the taxa that were not only abundant across experiments but also highly enriched in the ^15^N-labeled chitin compartment revealed a more selective group, indicative of a distinct microbial community associated with organic N mineralization. The taxa most associated with this compartment were *Flavihumibacter*, *Nonomuraea*, and unidentified members of Vampirovibrionaceae and Cyclobacteriaceae in Exp. 1; *Actinomadura*, *Kribbella*, *Nocardia*, *Nonomuraea* and *Streptomyces* in Exp. 2; and *Halomonas*, *Mycoplasma*, *Peptoniphilus*, *Promicromonospora*, *Thermosporothrix*, and unidentified Streptosporangiales and Cyclobacteriaceae in Exp. 3 (**Supplementary Table S6**).

It is also important to highlight the impact of inoculum complexity (gradient of size bottleneck imposed on the microbial inoculants) on the genus‐level distribution. Specifically, in more complex bacterial inocula, genera such as *Aquicella*, *Sporocytophaga*, *Flavisolibacter*, *Desmonostoc*, *Flavitalea*, *Hydrogenophaga*, and *Qipengyuania* were more abundant; as inoculum complexity decreased, we observed increased relative abundances of *Kribbella*, *Sphingopyxis*, *Rhodobacter*, and *Deinococcus*, indicating that consortium simplification favoured development of these taxa (**Supplemenaty Table S6**).

## 4 Discussion

### 4.1 Reduced microbial inputs into the root-free compartments limits ¹^5^N transfer to plants

In this study, we investigated the effect of bacterial community simplification and modes of microbial inoculation on ^15^N transfer to plants from organic source (chitin) placed beyond the direct reach of roots via the mycorrhizal pathway. In Experiments 1 and 2, microbial filtrates were inoculated into the compartments, in addition to the rhizosphere, to establish an active microbial community with the potential to degrade chitin and subsequently promote the transfer of N to the plant via the fungal network. In contrast, Experiment 3, in which no inoculation was applied to the compartments, tested whether AM hyphae acted as highways that enable bacteria to reach distant, nutrient-rich zones, and assessed the impact of this transport on the efficiency of N mineralization. Although size fractionation simplified the bacterial community, surprisingly it did not largely affect chitin mineralization, and AM hyphae alone were unable to transport decomposer microorganisms effectively into the root-free compartments. Rather, chitin mineralization — and the consequent N provision to plants — was substantially enhanced only when the microbiota were inoculated directly into the compartments amended with isotopically labeled chitin.

Recent studies consistently highlight cooperation between *R. irregularis* and the soil microbial community as a key component in the acquisition and transfer of ^15^N to plants (Hestrin et al., 2019; Rozmoš et al., 2022; Wang et al., 2024; Brandt et al., 2025; Vaishnav et al., 2025). However, our data suggest that the effectiveness of this process depends on the spatial context and the distribution of microorganisms in the soil. The beneficial effect of microbial communities on ^15^N mobilization and plant growth was observed between experiments, being most pronounced in Exps. 1 and 2, where microbial filtrate were present throughout the entire system, including the root-free compartments (Bukovská et al., 2021; **Figure 2A**). Contrary to our first hypothesis, we found no evidence that size fractionation of the microbial community affected the efficiency of ¹^5^N transfer to the plants. Instead, the observed variability likely reflects differences in the overall microbial activity within the root-free compartments rather than any specific effect of community structure. This finding contrasts with results reported by previous studies, which observed significant effects of microbial diversity on plant biomass and soil processes when working with field soils (Wagg et al., 2021; Romero et al., 2023, Romero et al., 2025). Such discrepancies may be explained by differences in the origin and complexity of the microbial inoculum: the use of field soil in the above experiments likely promoted a more taxonomically and functionally diverse community compared to our greenhouse-maintained pot cultures, which served as microbial inoculum source in our experiments. Moreover, in the previous studies, mycorrhizal fungi were probably excluded in the smaller size fractions during the sieving process, while in our experiment all treatments were equally inoculated with *R. irregularis.* This uniform mycorrhizal inoculation may have minimized potential contrasts among microbial fraction treatments.

Interestingly, we observed higher leaf N concentrations in mesocosms inoculated with more simplified microbial communities, and particularly when the microbes were only inoculated under the seeds in Exp. 3. However, the dilution of N within plant biomass did not necessarily lead to a decrease in total N uptake by the plant but rather reflected how N was distributed relative to increased plant biomass (Ata-Ul-Karim et al., 2017; Ciampitti et al., 2022). In other words, larger plants may have distributed the absorbed N in greater tissue mass, reducing its relative concentration per unit of biomass dry weight. In addition, the soil biota itself can act as an important temporary reservoir of N, so greater microbial abundance in soil may have delayed its immediate transfer to plant tissues due to the N immobilization in the living microbial biomass (Averill et al., 2015; Liu et al., 2016; Mason-Jones et al., 2022). This notion was consistent with our observation of lower N content in 90 and 1000 μm treatments in Exp. 2 as compared to the 2 μm treatment in the same experiment (analyses not shown).

### 4.2 Hyphae of *R. irregularis* play a limited role in bacterial migration between compartments

It is widely recognized that the extra-radical hyphae of AM fungi release exudates that modify the physical and chemical properties of the hyphosphere and recruit specialized microbial consortia potentially useful in organic-matter degradation, a process that is fundamental for releasing and acquisition of nutrients otherwise inaccessible to the plant or fungus alone. However, our findings largely diverged from the assumed scenario (Zhou et al., 2023; Anckaert et al., 2024; Duan et al., 2024; Jin et al., 2024). In the rhizosphere, the bacterial community remained remarkably similar across the different experiments, which was expected due to the continuous supply of root exudates (using the same host plant species) and the associated modifications to soil physicochemical parameters that select for a characteristic rhizosphere microbiome (Turner et al., 2013; Kuzyakov and Razavi, 2019; Ling et al., 2022). On the other hand, we observed marked differences generated by the diversity limitation or complete absence of microbial inoculation in the different root-free compartments.

In Exp. 3, in particular, we observed that the absence of bacterial inoculation into the root-free compartments not only compromised ^15^N mineralization but also appeared to modify AM hyphal growth patterns. Specifically, the hyphae were preferentially colonizing the mineral N-enriched compartments, likely as a strategy to access a readily assimilable N source (Sheldrake et al., 2018; Basiru et al., 2025). However, despite the apparent preference for colonization in certain compartments, consistent presence of inoculant *R. irregularis* was confirmed by qPCR in all experiments and compartments, meaning that the AM fungal hyphae were present in all compartments and their absence could not explain lack of efficient N transfer from chitin to plants in Exp. 3. Yet, in contrast to efficient AM fungal colonization, we were surprised by the low efficiency of bacterial migration along the hyphae towards the organic N, highlighting limitations in bacterial transport in this system. Only after inoculation of a very diverse microbial filtrate (passed through 1 mm mesh) into the rhizosphere in Exp. 3 did we observe an increase in bacterial colonization of the root-free compartment containing chitin and, consequently, a slight increase in the transport of ¹^5^N towards the plants. In this treatment, the high abundance of prokaryotic genera possibly associated with the N cycle, such as *Desmonostoc*, *Flavisolibacter*, *Flavitalea*, *Hydrogenophaga*, and *Qipengyuania*, suggests that either the intrinsic motility of some of these bacteria or the transport mediated by other organisms (e.g., protists) present in the more diverse filtrate favoured greater colonization of the chitin compartment and subsequent N mineralization (Chen et al., 2018; Gao et al., 2019; Wang et al., 2022; Almeida et al., 2023; Qiang et al., 2024; Flores and Herrero, 2010; Ilangumaran et al., 2024; Vieira et al., 2025). Yet, this process obviously was slow, it took weeks, and the generally low ^15^N transport from chitin to plants observed in Exp. 3 contradicted our second hypothesis to a large extent.

We acknowledge a methodological caveat regarding plant establishment across experiments. In Experiment 3, surface-sterilized seeds were used rather than pre-grown seedlings to preserve semi-sterile conditions; cylinders remained sealed throughout the experiment, which made seedling transplantation technically impractical. This approach should not have limited hyphae-mediated bacterial transport, as continuous root growth and the extended duration of Experiment 3 (9 weeks) may have facilitated microbial movement into the root-free compartments via rhizosphere expansion and AM hyphal networks. Although ^15^N transfer to plants was significantly reduced in Exp. 3 as compared to Exps. 1 and 2, N mineralization has not completely stopped, indicating some microbial activity even under such extreme conditions. This is probably due to the ability of some prokaryotes to migrate into the compartment and contribute to N mineralization. For instance, although scarce, studies suggest that *Halomonas* and *Mycoplasma* have motility mechanisms—flagellar and gliding, respectively, which would allow them to reach the chitin compartment (Miyata and Hamaguchi, 2016; Yin et al., 2022). Similarly, *Promicromonospora* and *Thermosporothrix*, through mycelial or filamentous growth, have also demonstrated to have the ability to colonize the distant soil patches (Yabe et al., 2016; Mohammadipanah et al., 2017). In the case of *Peptoniphilus*, a genus typically considered as non-motile, we speculate that its occurrence in the chitin compartment results from secondary transport, “hitchhiking” with motile bacteria, via actinobacterial mycelia, or through other vectors such as protists (Hawxhurst et al., 2023; Taerum et al., 2025). These distinct forms of motility, still poorly characterized in soil ecosystems, indicate that both active motility and passive transport mechanisms may act complementarily in the soil N dynamics.

Because the Exp. 3 was specifically devised to test if the microbes inoculated in a distance from the nutrient source were able to quickly migrate to the root-free compartments and fulfil the organic N mineralization function efficiently, microbial consortium in Exp. 3 was introduced exclusively into the rhizosphere. This spatially limited inoculation may have reduced the presence of keystone microorganisms in the chitin compartment, which were found in Exps.1 and 2. For example, *Flavihumibacter* and *Nonomuraea* in Exp. 1, and *Actinomadura*, *Kribbella*, *Streptomyces*, *Nocardia*, and *Nonomuraea* in Exp. 2, were present precisely in the contexts where N mobilization was most efficient. This strongly suggests that these microorganisms perform functionally relevant roles in the degradation of organic compounds, promoting the release of AM fungus-assimilable N forms, which is consistent with previous reports of *Streptomyces*, *Nocardia*, and *Nonomuraea* in chitin degradation (Usui et al., 1987; Hjort et al., 2009; Iwasaki et al., 2020). It is important to consider that distribution of these genera in the soil may be limited due to their reduced dispersal capacity, since several of them are considered immobile or have extremely reduced motility (Lee et al., 2014; Ozdemir-Kocak et al., 2017; Zhao et al., 2011). Among those, *Streptomyces* and *Actinomadura*, although capable of colonizing soil, depend solely on the extension of their filaments, which gradually penetrate the substrate from their initial establishment point, or on motile bacteria acting as transport vectors (Zhao et al., 2015; Muok et al., 2021). Thus, the presence or absence of these microorganisms in different compartments can directly influence N-cycling efficiency (Zhao et al., 2015; Muok et al., 2021) and this may depend on efficiency and rate of their long-distance dispersal in the microcosms inoculated patchily (such as in our Exp. 3). Accordingly, our results indicate that the AM fungal hyphae were not the primary dispersal vector responsible for the predominance of bacterial groups important in N mineralization in the ¹^5^N chitin-enriched compartment. Instead, it is more likely that their presence and activity resulted from direct inoculation of these communities into the chitin compartments in Exps. 1 and 2, thereby allowing colonization even by taxa with limited motility without reliance on secondary transport vectors. Although our experiments provided evidence of specialized microbial community potentially transported along *R. irregularis* hyphae, this mechanism proved inefficient to secure fast N mineralization in chitin compartments, and was further strongly conditioned by the presence of a sufficiently diverse and motile microbiota – which is largely in contrast to popular believes present in current literature (Kearns, 2010; Sharma et al., 2020; Jiang et al., 2021; Anckaert et al., 2024; He et al., 2024). Thus, our hypothesis that AM fungal hyphae act as vectors for bacteria specialized in degrading organic N sources was only partially supported, leaving the questions open about how fast and how efficient such processes could be and whether they would suffice plant nutrition of short living (annual) plans such as agricultural crops.

It is important to recognize limitations that may affect the robustness of our findings. First, the three experiments reported here were conducted sequentially due to logistical constraints (limited amount of PMMA cylinders, limited glasshouse size), which may have introduced subtle environmental variation among them and thus the interpretation requires caution when directly comparing their results. Additionally, we focused our analysis exclusively on the prokaryotic community, without assessing the communities of other microorganisms (such as protists or saprotrophic fungi), which could potentially interfere with or facilitate microbial dispersal. Similarly, physical properties of the potting soil (porosity, texture, aggregate distribution) likely influence AM hyphal architecture and, consequently, the efficiency of bacterial transport; future experiments that manipulate these edaphic characteristics may also identify conditions that favor or restrict fungus-bacteria interaction. Finally, incorporating different AM fungal species, whose extraradical foraging strategies differ markedly from *R. irregularis* will be essential in future studies to understand how the different fungi contribute to microbial dispersal in soils (Hart and Reader, 2005; Antunes et al., 2025). Yet it might be difficult to obtain such AM fungal inoculants in sufficient amounts and bacteria-free.

By employing a rich and diverse soil microbial community—more representative of natural conditions than *in vitro* assays or pot experiments with isolated microbial strains—our study demonstrated how bacterial diversity and intrinsic motility influenced the exploration and utilization of organic N, as well as the potential of AM fungal highways. These findings provide a foundation for future investigations into the design of microbial consortia that enhance nutrient cycling and promote sustainability in agricultural systems.

## 5 Conclusions

In summary, our results demonstrate that the efficiency of organic N mineralization and subsequent N uptake by plants via the mycorrhizal pathway was strongly conditioned by the prior presence of competent bacterial communities in organic N compartments. In the absence of these microorganisms, such compartments were not attractive for the AM fungal hyphae (as seen in Exp. 3, above all). The AM hyphal networks proved surprisingly little effective in transporting bacteria and inducing N mineralization in originally sterile compartments, even when a complex microbiome was introduced to the rhizosphere several centimeters away from the compartments. As a consequence, chitin mineralization and chitin-derived N transfer to plants were severely impaired as compared to scenario where the microbes were placed directly into the chitin compartments. In addition, microbial dispersal along the hyphae was slow and likely depended on both the intrinsic motility of the bacterial community and the diversity of the microbial inputs. These findings highlight the importance of integrating not only the taxonomic composition of microbial communities but also their functional traits and the physico-chemical features of the substrates/soils into future experimental designs, in order to more precisely elucidate the role of AM fungal highways in soil N dynamics and plant nutrient uptake efficiency.

## Data Availability

Primary data on which the analyses presented in this paper are all based, are provided as **Supplementary Materials**. Raw sequencing data are available in NCBI Sequence Read Archive (SRA) under Bioproject PRJNA1289392.

## Supplementary material

Supplementary Figure 1 Plants and experimental containers in the different experiments presented here. Appearance of pots with plants in Exp. 1 shortly before harvest **(A)**. Pre-cultivation of mycorrhiza-inoculated plant compartments with plants in Exp. 2 **(B)** and appearance of the large experimental pots in Exp. 2 after transferring the plant compartments **(C)**. Appearance of the different microbial inocula in Exp. 2 – from left to right, sieved through 1 mm, 90 μm, 32 μm, 5 μm and 2 μm sieves/filters **(D)**. Setting up pre-sterilized microcosms in Exp. 3 into the transparent cylinders for protecting them from external microbial inputs **(E)**. Experimental microcosms in Exp. 3 in the glasshouse at the beginning of the plant cultivation **(F)** and shortly before harvest **(I)**. Root-free compartments after removing them from the experimental pot in Exp. 3 **(G)** and after unscrewing the lid holding the 32 μm mesh (i.e., entrance to the buffer compartment, **(H)**. Colonization of *Andropogon gerardii* roots as revealed by Trypan blue staining and microscopy in Exp. 3 **(J)**.
