# Supplementary figures and images for: Low spatial mobility of associated microbes along the hyphae limits organic nitrogen utilization in the arbuscular mycorrhizal hyphosphere
Source: Front Plant Sci. 2026 Jan 12;16:1706684. doi: 10.3389/fpls.2025.1706684 (PMC12833381; doi:10.3389/fpls.2025.1706684)

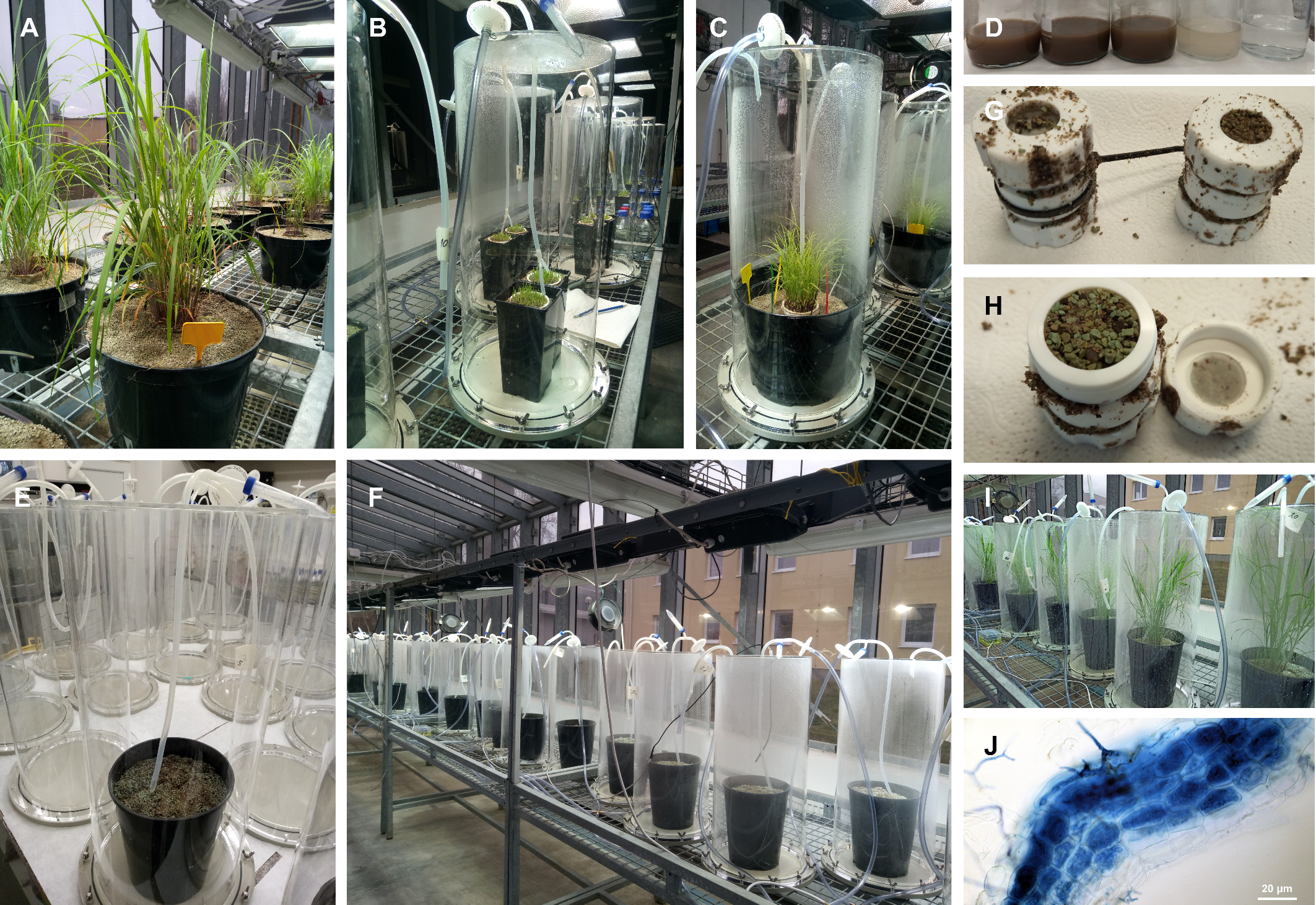

Supplement: Supplementary Figure 1 — Plants and experimental containers in the different experiments presented here. Appearance of pots with plants in Exp. 1 shortly before harvest (A). Pre-cultivation of mycorrhiza-inoculated plant compartments with plants in Exp. 2 (B) and appearance of the large experimental pots in Exp. 2 after transferring the plant compartments (C). Appearance of the different microbial inocula in Exp. 2 – from left to right, sieved through 1 mm, 90 μm, 32 μm, 5 μm and 2 μm sieves/filters (D). Setting up pre-sterilized microcosms in Exp. 3 into the transparent cylinders for protecting them from external microbial inputs (E). Experimental microcosms in Exp. 3 in the glasshouse at the beginning of the plant cultivation (F) and shortly before harvest (I). Root-free compartments after removing them from the experimental pot in Exp. 3 (G) and after unscrewing the lid holding the 32 μm mesh (i.e., entrance to the buffer compartment, (H). Colonization of Andropogon gerardii roots as revealed by Trypan blue staining and microscopy in Exp. 3 (J). [file Image1.jpeg]
